# Internship workplace preferences of final-year medical students at Zagreb University Medical School, Croatia: all roads lead to Zagreb

**DOI:** 10.1186/1478-4491-4-7

**Published:** 2006-04-01

**Authors:** Ozren Polasek, Ivana Kolcic, Aleksandar Dzakula, Mario Bagat

**Affiliations:** 1 Department of Medical Statistics, Epidemiology and Medical Informatics, Andrija Stampar School of Public Health, Medical School, University of Zagreb, Zagreb, Croatia; 2 Public Health Sciences, University of Edinburgh, Edinburgh, UK; 3 Department of Social Medicine and Health Care Organization, Andrija Stampar School of Public Health, Medical School, University of Zagreb, Zagreb, Croatia; 4 Croatian Institute for Health Insurance, Zagreb, Croatia

## Abstract

**Background:**

Human resources management in health often encounters problems related to workforce geographical distribution. The aim of this study was to investigate the internship workplace preferences of final-year medical students and the reasons associated with their choices.

**Method:**

A total of 204 out of 240 final-year medical students at Zagreb University Medical School, Croatia, were surveyed a few months before graduation. We collected data on each student's background, workplace preference, academic performance and emigration preferences. Logistic regression was used to analyse the factors underlying internship workplace preference, classified into two categories: Zagreb versus other areas.

**Results:**

Only 39 respondents (19.1%) wanted to obtain internships outside Zagreb, the Croatian capital. Gender and age were not significantly associated with internship workplace preference. A single predictor variable significantly contributed to the logistic regression model: students who believed they would not get the desired specialty more often chose Zagreb as a preferred internship workplace (odds ratio 0.32, 95% CI 0.12–0.86).

**Conclusion:**

A strong preference for Zagreb as an internship workplace was recorded. Uncertainty about getting the desired specialty was associated with choosing Zagreb as a workplace, possibly due to more extensive and diverse job opportunities.

## Introduction

Human resources are a central issue in health system planning [1], and a major concern for both developed and developing countries [2]. Smaller, mid-income countries that do not rely on immigrant health workers depend on their own resources in producing a qualified health workforce. In such countries, careful balancing of the demand and supply of health care professionals is very important. This involves estimating the numbers of health care professionals who must be educated in order to offset losses due to causes such as retirement, death, disability, emigration and leaving the medical profession. Special attention must also be given to other elements, such as demographic change and secular trends, often requiring re-evaluation and re-balancing of the workforce. In its essence, good human resource management depends on the collection of reliable, evidence-based information [2]. Secondly, health care systems in mid-income countries require that all valuable human resources be carefully distributed among the desired and less-desired areas of the country.

Almost all health systems experience various levels of geographical distribution problems [3]. Most physicians and other health professionals place a high value on urban or capital areas as preferred workplaces. As a consequence, rural and remote areas are often under-resourced in terms of skilled manpower, presenting a challenging problem in the planning and quality of delivered health care in those areas. Despite a proliferation of initiatives and techniques aimed at attracting physicians to less desirable areas, no "magic bullet" has yet been found [4]. Recruitment of students from a rural background has offered some success in attracting and retaining rural physicians [5].

Geographical disparities in the distribution of medical staff in Croatia have long been recognized [6]. The situation was further complicated by the war and the reform of the health care system, resulting in the decline of the physician-to-population ratios [7].

Some problems in the health care system were reflected by medical students, as well. A recent study on final year medical students' specialty preferences classified the reasons for specialty choice into five categories – ambition, compassion, quality of life, resignation and confusion [8]. The last two responses represented almost a fifth of all responses, indicating a substantial degree of confusion and resignation among students when considering a future specialty [8]. These findings, along with the high percentage of students considering emigration [8], indicate a certain level of dissatisfaction with the current health care system among graduating medical students.

The aim of this study was to investigate internship workplace preferences of final-year medical students at Zagreb University Medical School, and to determine factors associated with the internship workplace preference.

## Subjects and methods

### Design

We analysed final-year Zagreb University Medical School students' internship workplace preference and factors associated with their decision. Students were surveyed during autumn 2004, several months before their graduation.

### Setting

The medical education system in Croatia is based on a six-year undergraduate curriculum offered in four medical schools (Zagreb, Rijeka, Osijek and Split). After graduation, students undertake a one-year internship. The availability and location of each internship post are publicly announced, and physicians can apply for any post they find attractive. After application, physicians are interviewed and scored on the basis of undergraduate academic record (grade-point average, length of study, scientific involvement, etc.) and extracurricular activities. The best-ranked applicants are accepted and start working as interns.

During internship, interns must complete 18 rotations in major medical subjects (both clinical and public health). The interns choose the sequence of rotations, while the Ministry of Health and Welfare defines the duration and programme of each rotation. On completion of the internship, physicians are required to take a registration exam, after which they can register at the Croatian Medical Chamber and practise medicine in Croatia.

After obtaining their licence, physicians can compete for a residency (specialization). A similar scoring system is applied, again favouring undergraduate academic performance.

Analysis of the physician market in Croatia exhibits a significant decrease in the number of physicians employed in government-owned health facilities [9]. At the same time, a gradual increase in the number of physicians in rented offices (that have a contract with the Croatian Institute for Health Insurance) was recorded. The number of physicians employed in privately-owned facilities exhibited no long-term trend [9]. The overall number of unemployed physicians has been declining in recent years, and combined with the new maximum working hours scheme, suggests a possible workforce shortage [10].

### Subjects

Final (sixth)-year medical students were recruited for this study. Respondents were then divided into two groups: Zagreb residents (born and living in Zagreb) and non-Zagreb residents (from other parts of Croatia and other countries).

### Measurements

The study was based on a survey consisting of 18 questions. Questions were grouped in five sections: (1) general data (sex, grade-point average, place of birth and age); (2) preferred specialties and belief as to whether they would get the desired specialty; (3) scientific involvement questions (involvement in scientific projects, time-frame, publication output); (4) emigration preferences (whether a student was considering emigration, why, and what he or she expected); and (5) internship workplace preferences. Surveys that did not contain sufficient information or contained intentionally misleading answers were excluded from the study.

## Analysis

Data were analysed using both bivariable and multivariable methods. The Chi-square test was used in the analysis of categorical data. The Mann-Whitney test was used to analyse students' age, due to a non-normal data distribution. Logistic regression was used to predict factors underlying the internship workplace preferences. Several academic parameters were used as predictor variables: grade-point average, failure to pass one or more undergraduate year(s), students' beliefs that they would get the desired residency (specialty), readiness to emigrate, and scientific involvement measured through active involvement in research projects. Statistical analysis were performed with SPSS software, version 12.0.0 (SPSS Inc., Chicago, IL, USA), with statistical significance set at *P *< 0.05.

## Results

The final sample consisted of 204 surveys (out of 240 students: response rate 85.0%). A total of 81 students were Zagreb residents (39.7%). Of the remainder, 106 students were non-Zagreb resident Croatians (52.0%), while 17 (8.3%) were not born in Croatia. Most of these (15; 88.2%) were born in the neighbouring Bosnia and Herzegovina.

A total of 140 (68.6%) students preferred to work in Zagreb, while 25 (12.3%) students expressed uncertainty about their internship workplace preference (Table 1). Among the latter group, seven (3.4%) students responded with a high level of resignation, saying that they would work "wherever they got paid for it", or saying that they would leave medicine. There was no significant gender difference (χ^2 ^= 1.42, d.f. = 1, *P *= 0.234) or age difference (Mann-Whitney Z = -0.70, *P *= 0.485) in workplace preference. A comparison of the grade-point average between students preferring Zagreb versus other parts of Croatia did not exhibit significant differences (Zagreb median 3.8, interquartille range 0.6; other parts median 3.6, interquartile range 0.6, Mann-Whitney Z = -1.35, *P *= 0.176).

**Table 1:** Residence and workplace preference among final-year medical students at Zagreb University Medical School, academic year 2004–2005.

**Workplace preference**	**Residence**		**Total**
		
	**Zagreb N (%)**	**Other N (%)**	
Zagreb	59 (72.8)	81 (65.9)	140 (68.6)
Other	13 (16.1)	26 (21.1)	39 (19.1)
Not sure/Don't know	9 (11.1)	16 (13.0)	25 (12.3)
Total	81 (100.0)	123 (100.0)	204 (100.0)

A total of 128 students (62.7%) believed they would secure the desired specialty, exhibiting no gender differences (χ^2 ^= 0.01, d.f. = 1, *P *= 0.907). A total of 96 (68.6%) students preferring internship in Zagreb believed they would secure the desired residency post, compared to 32 (82.1%) students preferring to work outside Zagreb (χ^2 ^= 2.72, d.f. = 1, *P *= 0.100).

A logistic regression model with internship workplace preference as the outcome variable was created. The model provided a good fit for the data (Hosmer and Lemeshow test *P *= 0.898). The only variable significantly associated with internship workplace preference was the student's belief that he or she would secure a residency in the desired specialty (Table 2).

**Table 2:** Logistic regression model predicting workplace preference among final-year Zagreb University Medical School students, academic year 2004–2005.

**Predictor**	***P***	OR (95% CI*)
***Sex***		
Male	0.425	Ref.
Female		0.69 (0.28–1.70)
***Age***	0.815	1.03 (0.80–1.34)
***Grade-point average***	0.127	2.40 (0.78–7.42)
***Ever failed a study year***		
No	0.170	Ref.
Yes		1.97 (0.75–5.19)
***Believed they would secure the desired specialty***		
No	0.024	Ref.
Yes		0.32 (0.12–0.86)
***Readiness to emigrate***		
No	0.704	Ref.
Yes		1.19 (0.49–2.86)
***Involved in scientific work as a student***		
No	0.132	Ref.
Yes		2.51 (0.76–8.34)
***Interest in scientific work in the future***		
No	0.255	Ref.
Yes		1.73 (0.67–4.45)

*CI – confidence intervals

## Discussion

This study exhibited a strong preference for an internship workplace in Zagreb, the Croatian capital. A little less than 20% of students wanted to work outside Zagreb, where as much as three-fourths of the Croatian population lives.

The undergraduate medical curriculum of Zagreb University Medical School provides an opportunity for students to observe medical care provision in a remote setting. During the fourth curriculum year, students attend a one-week course "Community Practice". They are accommodated in a remote setting and introduced to elements of working in primary and secondary health care. Studies of similar programmes of longer duration have been positively associated with a preference for working in rural areas [11]. However, evaluation of the impact of this course at Zagreb University Medical School remains unavailable.

Analysis of the labour market exhibited a high interest in large urban areas (Zagreb, Rijeka, Osijek and Split), without vacant internships [10], indicating very high demand for any available position. The situation persists despite a large surplus of the potential internship positions on the national level, compared to the number of newly graduated physicians [10].

Compared to other parts of Croatia, Zagreb offers much broader long-term career possibilities, such as research fellowships at various academic institutions offered every year. These fellowships involve up to a six-year research period, during which a fellow is required to obtain a PhD degree, and in the case of clinical and some public health attachments, also specialize in the medical field related to the fellowship post.

Besides strictly medical careers, various careers related to medicine are more common in Zagreb. These include positions as sales representatives for medical equipment suppliers or provision of various other services related to medicine (e.g. as a part of the health Internet portal team, or telephone advisory services). Careers in the pharmaceutical industry have been very popular in recent years, with as many as 9% of final-year medical students considering this as a possible career option and 3% of students interested only in the pharmaceutical industry as a permanent career [8].

Because there are so many attractive alternative career options available to medical graduates in Zagreb, those who do not believe they will secure a residency in the specialty of their choice may choose to stay in Zagreb in the hope of finding a lucrative alternative career. An investigation of the medical graduates cohort might provide useful information on the proportion of young doctors who actually become employed in the health system and start working as physicians.

The findings of this study have rather depressing implications for the Croatian health and medical education systems. The fact that only 63% of students believed they would secure the desired specialty is related to a substantial level of dissatisfaction among students. This is consistent with the findings of the previous study, which found a great deal of confusion and resignation among final-year medical students [8]. Depletion of the health workforce through emigration presents another possible danger to the Croatian health system. Although during the past few years a very low emigration rate among physicians has been recorded [12], the possibilities for emigration after Croatia joins the European Union are far more alarming [13].

The question of the Croatian health workforce balance has recently become a subject of substantial public interest. The physician market in Croatia has gradually changed over the last few years. The number of unemployed physicians declined from 384 registered in the year 2000, to 72 in the year 2005 [10,14]. The same reports [10,14] indicated a serious projected shortage of senior medical staff in the years to come. Shortfalls of 398 consultants in internal medicine and 340 consultants in surgery are expected to occur by the year 2007.

The combination of the centralized workplace preferences, projected physician shortage and the possibility of an increased emigration rate after membership in the European Union could negatively affect health care provision in underserved areas, particularly remote areas affected by the recent war and remote mountainous and Adriatic island regions.

A shortcoming of this study is the use of data solely from Zagreb University Medical School. The situation in other medical schools might be different. Nevertheless, the study clearly demonstrates a strong tendency for students from Zagreb University Medical School to choose Zagreb as their desired internship workplace. This is associated with a widespread belief among students that they are unlikely to secure the residency of their choice. With a potential shortage of physicians on the horizon, fuelled by the possibility of emigration after Croatia enters the European Union, the Croatian health care and medical education systems require a thorough evaluation and a careful projection of health workforce demands.

## Competing interests

The author(s) declare that they have no competing interests.
